# Distinct Pathway of Human T-Cell Leukemia Virus Type 1 Gag Punctum Biogenesis Provides New Insights into Enveloped Virus Assembly

**DOI:** 10.1128/mBio.00758-18

**Published:** 2018-09-04

**Authors:** John P. Eichorst, Yan Chen, Joachim D. Mueller, Louis M. Mansky

**Affiliations:** a Institute for Molecular Virology, University of Minnesota, Minneapolis, Minnesota, USA; b Division of Basic Sciences, School of Dentistry, University of Minnesota, Minneapolis, Minnesota, USA; c School of Physics and Astronomy, University of Minnesota, Minneapolis, Minnesota, USA; d Masonic Cancer Center, University of Minnesota, Minneapolis, Minnesota, USA; Albert Einstein College of Medicine

**Keywords:** Gag, deltaretroviruses, oligomerization, retroviruses, virus assembly

## Abstract

The assembly of virus particles is a crucial aspect of virus spread. For retroviruses, the Gag polyprotein is the key driver for virus particle assembly. In order to produce progeny virus, once Gag is translated, it must translocate from the location in the cytoplasm where it is synthesized to the plasma membrane and form an oligomeric lattice that results in Gag puncta. The biogenesis of mature Gag puncta can trigger the budding process, resulting in virus particle production. While some aspects of the dynamics of Gag oligomerization and particle biogenesis have been observed with human immunodeficiency virus type 1 (HIV-1), the process of Gag punctum biogenesis remains poorly understood, particularly for other retroviruses. Here, we have conducted the most detailed studies thus far on Gag punctum biogenesis for human T-cell leukemia virus type 1 (HTLV-1). Using mEos2 photoconvertible fluorescent proteins and total internal reflection fluorescence microscopy (TIRF), we have found that HTLV-1 Gag was recruited to Gag puncta primarily from the plasma membrane. This was in stark contrast to HIV-1 Gag, which was recruited from the cytoplasm. These observations imply fundamental differences among retroviruses regarding the orchestration of Gag punctum biogenesis, which has important general implications for enveloped virus particle assembly.

## Abstract (precis)

This report describes the results of experiments examining the pathway by which the human retroviral Gag protein is recruited to sites along the inner leaflet of the plasma membrane where Gag punctum biogenesis occurs. In particular, clever and sensitive experimental methods were devised to image in living cells fluorescently labeled Gag protein derivatives from human T-cell leukemia virus type 1 (HTLV-1) and human immunodeficiency virus type 1 (HIV-1) at the plasma membrane. The photoconvertible fluorescent protein mEos2 was strategically utilized, as the fluorescence emission of Gag at the plasma membrane could be differentiated from that of cytosolic Gag. This experimental strategy allowed for the determination of the Gag recruitment pathway into Gag puncta. For HTLV-1 Gag, puncta recruited Gag primarily from the plasma membrane, while HIV-1 Gag was recruited from the cytoplasm. These observations represent the first report of HTLV-1 particle biogenesis and its contrast to that of HIV-1. The observed differences in the Gag recruitment pathways used by HTLV-1 and HIV-1 Gag provide key information that is useful for informing the discovery of novel targets for antiretroviral therapies directed at eliminating virus infectivity and spread.

## INTRODUCTION

A common feature among enveloped viruses that assemble at the plasma membrane is the requirement for membrane accumulation of viral structural proteins and nucleic acid in order for virus particle budding and release to occur. The general strategies used by enveloped viruses to accomplish this task are poorly understood. For retroviruses, such as human immunodeficiency virus type 1 (HIV-1), the Gag polyprotein is known to be the primary driver for virus particle assembly (1). Upon translation of the genomic RNA in the cytoplasm, Gag is translocated to a virus budding site at the plasma membrane where oligomeric Gag engages the cellular ESCRT (endosomal sorting complex required for transport) machinery and results in virus budding.

While HIV-1 has become the most extensively studied retrovirus in this regard, it is uncertain how representative the observations with HIV-1 are among retroviruses, including those of other human retroviruses such as human T-cell leukemia virus type 1 (HTLV-1). HTLV-1 infects about 15 million to 20 million individuals worldwide and is the etiological agent of an adult T-cell leukemia/lymphoma (ATLL) (2) as well as an inflammatory disease syndrome called HTLV-1-associated myelopathy (HAM)/tropical spastic paraparesis (TSP) (3–5). Early studies of HTLV-1 antibody prevalence rates showed a variance among geographical areas, ranging from 0.2 to 10% among adults (6). This antibody prevalence was found to increase with age, and it could affect as much as 20 to 50% of the female population aged 60 and above (6). Historically, HTLV-1 has been notorious for being difficult to study in cell culture, which has prohibited rigorous analysis of how these viruses replicate in cells, including the steps involved in retrovirus assembly. The details of how retrovirus particle assembly occurs are poorly understood even for other more tractable retroviral systems like that of HIV-1. Our understanding of retrovirus assembly, release, and maturation has advanced significantly in recent years, and this field has been catapulted by developments in imaging technology, structural biology, and cell and molecular biology. This increase in basic knowledge has already led to the application of developing novel inhibitors designed to target various aspects of virus assembly and maturation (7).

Though retroviral Gag proteins have relatively limited amino acid sequence homology, they do share structural similarities among the domains of Gag (8–10). Gag is a polyprotein and is composed of multiple domains, including the matrix (MA), capsid (CA), and nucleocapsid (NC) domains. HIV-1 Gag also possesses the p6 domain. The MA domain possesses the key determinants for membrane interaction and association with host tRNA and facilitating psi RNA binding specificity by Gag (11, 12). The CA domain typically encodes the prime determinants for Gag oligomerization which creates a lattice structure at the plasma membrane and forms a punctum that can be a productive site of virus particle release. The NC domain encodes amino acid determinants that interact with the viral RNA packaging signal.

It is well known that HTLV-1 is efficiently transmitted via cell-to-cell contacts, i.e., the virological synapse (VS) (for reviews, see references 13 and 14). This is also likely to be the case for HIV-1, but it has been commonly underappreciated. Cell-to-cell transmission is regarded as a major mechanism of HTLV-1 cell spread (15). Two types of cell-cell contacts via the formation of a VS can result in HTLV-1 transmission—polarized virus particle release into the VS and cell surface transfer of viral biofilms (16). Both modes rely on HTLV-1 Gag targeting to the points of cell contact and subsequent oligomerization and particle biogenesis and release. Irrespective of the HTLV-1 transmission route, virus particles are transmitted through the VS in confined areas protected from the immune response of the host (17). A remodeling of the cytoskeleton has also been implicated as a requirement for efficient virus transmission, along with the polarization of the microtubule organizing center (MTOC) inside the infected cell toward that of the permissive target cell. Previous studies have implicated the HTLV-1 Tax protein in enhancing adhesion protein (intercellular adhesion molecule 1 [ICAM-1]) expression in infected T cells in contact with uninfected T cells (18, 19), and this interaction induces cytoskeletal reorganization in the infected cell. Viral proteins, including the HTLV-1 Gag protein, have been found in the center of the VS and surrounded by adhesion proteins (19). “Viral biofilms” have been observed in the VS and are associated with an extracellular matrix (16); these biofilms could arise from continued particle production into the VS.

To date, the nature of the infectious form of HTLV-1 that is transmitted has not been thoroughly investigated. However, it seems most probable that infectious spread relies on the release of particles into the VS. In support of this, electron microscopy has revealed that HTLV-1 particles were present in the synaptic cleft between infected and uninfected lymphocytes (20). An understanding of how Gag punctum biogenesis occurs would be an important aspect of understanding how virus particles are assembled and formed, as well as providing key information for how infectious virus spread occurs via the VS.

Retrovirus particle biogenesis, for most retroviruses, requires the assembly, maturation, and release at the inner leaflet of the plasma membrane, followed by a viral proteolysis step that takes place after the particle has been released (21, 22). The time required for virus particle biogenesis to occur can be quite rapid. For example, the time necessary for HIV-1 particles to be formed and released varies based upon reports in the literature from 2 to 30 min (23–26).

Our recent studies have demonstrated that HTLV-1 Gag is capable of membrane targeting as a monomer, that HTLV-1 particle assembly can take place at low (i.e., nanomolar) cytoplasmic concentrations, and that there is a critical threshold concentration (approaching micromolar) prior to the observation of HIV-1 Gag associated with the plasma membrane (27). Additionally, previous observations suggested that HIV-1 Gag is recruited to Gag puncta from the cytoplasm (25, 28), although the precise dynamics of membrane recruitment were not elucidated.

In this study, we report new observations that point to novel features of HTLV-1 assembly not seen with HIV-1 or other retroviruses (29). In particular, we have used total internal reflection fluorescence microscopy (TIRF) (30) to study, in parallel, HTLV-1 and HIV-1 Gag punctum biogenesis. We employed HTLV-1 and HIV-1 Gag derivatives tagged with the mEos2 photoconvertible fluorescent proteins (31) to demonstrate that HTLV-1 Gag was recruited to Gag puncta primarily from the plasma membrane, while HIV-1 Gag was recruited to growing puncta from the cytoplasm. These striking differences in the biogenesis of Gag puncta provide one line of experimental evidence that differences exist in the virus particle assembly pathway among retroviruses—even closely related human retroviruses—which implies that distinct mechanisms can be utilized to ensure efficient virus particle assembly, which has general implications for enveloped viruses where virus particle assembly occurs at the plasma membrane.

## RESULTS

### Selective photoconversion of Gag-mEos2 at the plasma membrane.

**FIG 1:**
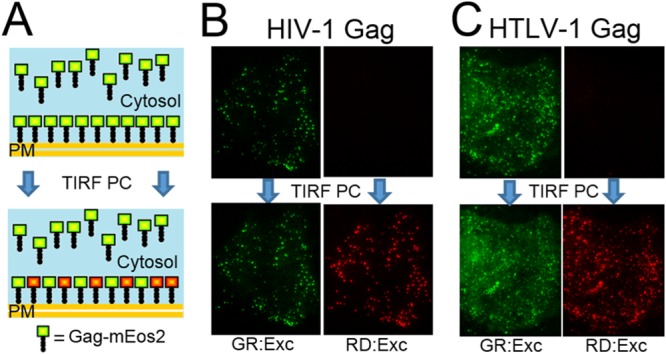
HTLV-1 Gag-mEos2 and HIV-1 Gag-mEos2 in living cells. (A) Schematic depiction illustrating the selective photoconversion (PC) of Gag-mEos2 at the plasma membrane (PM). (B) TIRF images taken with 488-nm excitation (green excitation [GR:Exc]) and 561-nm (red excitation [RD:Exc]) of a cell expressing HIV-1 Gag-mEos2 before PC (top) and after PC (bottom). (C) TIRF images of cells expressing HTLV-1 Gag-mEos2 prior to PC (top) display features of the cell near the plasma membrane for 488-nm excitation only. After PC (bottom), bright puncta and diffuse fluorescence along the plasma membrane are visible in images collected with both 488-nm and 561-nm excitation. Because of the large range of intensities in these images, the color scales of the images in panels B and C were computationally saturated. The same scale was maintained before and after PC for each imaging condition to facilitate direct comparison.

In order to examine the recruitment of Gag to Gag puncta (i.e., potential virus budding sites), the Gag-mEos2 (Gag protein labeled with the photoconvertible fluorescent protein mEos2) expressed in live cells was monitored by changing the color of their fluorescence emission from green to red exclusively at the inner leaflet of the plasma membrane. The photoconversion of Gag-mEos2 at the basal plasma membrane was conducted with incident light at 405 nm delivered by TIRF illumination. The evanescent field of TIRF restricts the activating light to the immediate vicinity of the glass/cell interface, resulting in efficient photoconversion at the basal plasma membrane, while cytosolic Gag-mEos2 remains in the green-emitting form (Fig. 1A). It has been shown that only ~60% of the mEos2 molecules photoconvert from the green state to the red state (32, 33). Consequently, TIRF illumination with 405-nm light results in a mixture of green- and red-emitting Gag-mEos2 at the basal plasma membrane (Fig. 1A). The green-emitting and red-emitting forms of mEos2 are experimentally distinguished by exciting the sample with two distinct wavelengths. Incident light at 561 nm selectively excites the photoconverted red-emitting form of mEos2, while 488-nm light predominantly excites the green-emitting form of mEos2 with some minor coexcitation of the mEos2 red-emitting form.

TIRF images of live cells transfected with HIV-1 Gag-mEos2 collected prior to photoconversion revealed puncta that were excited at 488 nm, but not at 561 nm (Fig. 1B, top panels), indicating that these puncta carry only green-emitting mEos2. Subsequent TIRF images of the same cell were taken after photoconversion identified puncta excited at 561 nm (Fig. 1B, bottom panels), reflecting the presence of the converted red form of mEos2. These puncta were also excited at 488 nm (Fig. 1B, bottom panels), consistent with the presence of a mixture of green- and red-emitting mEos2.

The epifluorescence image (excitation, 488 nm) of the same cell collected before the photoconversion step revealed in addition to the punctate fluorescence a diffuse background, which is caused by green-emitting, cytosolic HIV-1 Gag-mEos2 (see Fig. S1A, left panel, in the supplemental material). In contrast, the only fluorescence excited at 561 nm is the autofluorescence located near the cell nucleus (Fig. S1A, right panel). After photoconversion, bright puncta appeared along with autofluorescence in the epifluorescence image acquired with 561-nm excitation (Fig. S1B). By subtracting the epifluorescence image in Fig. S1B from the epifluorescence image in Fig. S1A, right panel, we eliminated the autofluorescence contribution (Fig. S1C). The absence of diffuse fluorescence in the difference image confirmed the absence of photoconverted HIV-1 Gag-mEos2 in the cytoplasm (Fig. S1C), which demonstrates that TIRF illumination at 405 nm achieves selective activation of Gag-mEos2 at the basal plasma membrane.

10.1128/mBio.00758-18.1 FIG S1  Epifluorescence images of cells expressing HIV-1 Gag-mEos2. (A) Prior to photoconversion (PC), the epifluorescence image displays diffuse cytosolic fluorescence and puncta upon excitation at 488 nm (left panel). Epifluorescence image of the same cell excited at 561 nm before PC (right panel) shows only autofluorescence near the nucleus. (B) After PC, bright puncta as well as the autofluorescence appear in the epifluorescence image excited by 561-nm light. (C) The images in the right panel of panel A and the left panel of panel B were subtracted to create this difference image. The lack of diffuse fluorescence between the puncta confirms the absence of cytosolic activation. Because of the large intensity range present in these images, the color scale of the images was computationally saturated to visualize both dim and bright features. Download FIG S1, TIF file, 1.6 MB. Copyright © 2018 Eichorst et al. 2018 Eichorst et al. This content is distributed under the terms of the Creative Commons Attribution 4.0 International license.

TIRF images of HTLV-1 Gag-mEos2 before photoconversion observe puncta only with 488-nm excitation (Fig. 1C, top panels), which confirms that these puncta contain unconverted mEos2. The diffuse fluorescence located between the puncta (Fig. 1C, right panel, bottom) is likely being emitted by nonpunctate HTLV-1 Gag-mEos2 associated with the basal plasma membrane (referred to as nonpunctate Gag). Following the photoconversion process, puncta appeared in the image collected with 561-nm excitation (Fig. 1C, bottom panels), indicating that a color change has taken place. These puncta were also excited at 488 nm (Fig. 1C, bottom panels), because the photoconverted puncta possess a mixture of green-emitting and red-emitting HTLV-1 Gag-mEos2.

### Analysis of Gag-Eos2 photoconversion.

As shown in Fig. 1, photoconversion leads to substantial changes in the color of fluorescence as detected by excitation at 488 nm and 561 nm. To quantify these differences, we have defined the green intensity fraction as *α*_*G*_ = *I*_488_/(*I*_488_ + *I*_561_) where *I*_488_ and *I*_561_ represent the intensities of a punctum excited at 488 nm and 561 nm, respectively. The limiting values of *α*_*G*_ before and after photoconversion were determined experimentally. A histogram of *α*_*G*_ from puncta containing unconverted HIV-1 Gag-mEos2 peaked around a value of 0.9 (Fig. S2). This value is lower than the theoretical limit of 1 because of small background contributions from the image excited at 561 nm.

10.1128/mBio.00758-18.2 FIG S2  Analysis of the extent of the green intensity fraction of mEos2 prior to photoconversion. (A) Cells transfected with HIV-1 mEos2-Gag and unlabeled HIV-1 Gag were imaged in the TIRF mode with 488-nm excitation before photoconversion (PC). This image is displayed using a computational color scale so that both the dim and bright puncta are simultaneously visible. (B) The green intensity fraction of the puncta from panel A was displayed as a histogram with a peak at ~0.9 before PC. Download FIG S2, TIF file, 2 MB. Copyright © 2018 Eichorst et al. 2018 Eichorst et al. This content is distributed under the terms of the Creative Commons Attribution 4.0 International license.

In order to establish the values of *α*_*G*_ corresponding to the maximum extent of photoconversion, an incremental number of pulses of 405-nm light were incident on a cell containing HIV-1 Gag-mEos2 proteins (Fig. S3). Intermittently, TIRF images (Fig. S3A and B) were taken of the fluorescence excited at 488 nm and 561 nm to monitor the green intensity ratio *α*_*G*_ of the puncta. The mean and standard deviation of α_*G*_ as a function of the number of 405-nm pulses were determined (Fig. S3C). The green intensity fraction α_*G*_ steadily decreased and ultimately approached a terminal value of ~0.3 after 500 pulses, indicating that the maximum extent of photoconversion was reached. The overall fluorescence intensity of the images collected with 561-nm excitation rose with exposure to the pulsed 405-nm light (Fig. S3B), reflecting the increase in the number of photoconverted mEos2 proteins. In contrast, there is a decrease in the fluorescence intensity of the corresponding images collected with 488-nm excitation (Fig. S3A). The remaining fluorescence at this excitation wavelength is caused by residual coexcitation of mEos2 in the red-emitting state and the presence of a pool of mEos2 that cannot convert into the red-emitting form.

10.1128/mBio.00758-18.3 FIG S3  Analysis of the maximum extent of Gag-mEos2 photoconversion. (A) Cells expressing HIV-1 Gag-mEos2 were exposed to 20-ms pulses of 405-nm light in the TIRF mode. Images of the cell taken in the TIRF mode with 488-nm excitation (A) and 561-nm excitation (B) after applying a specific number of pulses. (C) The green intensity fraction of Gag-mEos2 puncta was calculated from the TIRF images. The mean and standard deviation (error bar) of the green intensity fraction are plotted as a function of the number of 20-ms pulses of 405-nm light. All images are displayed using a linear color scale. The image series of each panel uses a fixed intensity scale to facilitate visual comparison. Download FIG S3, TIF file, 1.1 MB. Copyright © 2018 Eichorst et al. 2018 Eichorst et al. This content is distributed under the terms of the Creative Commons Attribution 4.0 International license.

These results establish the experimentally accessible range of the green intensity fraction which spans from ~0.3, representing the full extent of photoconversion of mEos2, to values near 0.9, reflecting entirely unconverted mEos2 proteins. All experiments in this study used conditions that resulted in maximal photoconversion of Gag-mEos2 at the basal plasma membrane.

It should be noted that the incident violet light used to photoconvert mEos2-Gag could impact cell viability. To address this, cells were examined with a blue nuclear dye (NucBlue) after photoconversion. Nonviable cells become permeable to this dye. Cells transfected with both HIV-1 Gag-mEos2 and HTLV-1 Gag-mEos2 were examined after photoconversion by using NucBlue. Analysis of cells 1 h after photoconversion revealed no apparent NucBlue staining, which was interpreted as exposure to the incident violet light having no significant impact on cell viability. In particular, no cell nuclei that possessed violet fluorescence 25 min following photoconversion were observed. For a control, ethanol was added to cells, which allowed NucBlue to be readily observed in cell nuclei (Fig. S4), confirming that the cells were not viable.

10.1128/mBio.00758-18.4 FIG S4  Analysis of cell viability. (A) The HTLV-1 Gag-mEos2 proteins present at the plasma membrane were terminally photoconverted and imaged with TIRF using 488-nm excitation. The outline of the cell is plotted in white on this image. (B and C) Following photoconversion, NucBlue was added to cell culture medium, and the cell was monitored with epifluorescence and 405-nm excitation for approximately 25 min. (D) Efficient visualization of cell death was confirmed by the addition of ethanol to cell culture medium, which resulted in nuclei with prominent violet fluorescence. Download FIG S4, TIF file, 1.1 MB. Copyright © 2018 Eichorst et al. 2018 Eichorst et al. This content is distributed under the terms of the Creative Commons Attribution 4.0 International license.

### Stability of the photoconverted Gag-mEos2 proteins at the plasma membrane.

We conducted measurements to determine whether the color of the nonpunctate Gag-mEos2 present at the plasma membrane changes over a period of 25 min after photoconversion. In these experiments, HTLV-1 Gag-mEos2 proteins present at the cell’s plasma membrane were terminally photoconverted with a series of short pulses of 405-nm light in TIRF mode. Following the photoconversion step, the intensity of the nonpunctate (diffuse) fluorescence signal was detected with TIRF-based imaging and quantified at selected time points (Fig. S5). Because the nonpunctate signal is faint, a large region of interest was chosen to provide sufficient averaging for computing the mean intensity. During that time interval, only minor changes in the intensity of diffuse red fluorescence located between the puncta were observed, likely indicating that the photoconverted HTLV-1 Gag-mEos2 proteins remained distributed on the plasma membrane during the time scales studied in this report.

10.1128/mBio.00758-18.5 FIG S5  Intensity of nonpunctate, photoconverted HTLV-1 Gag-mEos2 at the plasma membrane. (A) TIRF images of a HeLa cell with HTLV-1 Gag-mEos2 were collected at the indicated time points before and after photoconversion with 488-nm excitation. (B) The same cell in panel A was imaged with TIRF using 561-nm excitation at the indicated time points before and after photoconversion. (C) The average intensity from the regions of interest outlined in white in images are plotted over time. No green intensity fraction was calculated, because the low intensity from nonpunctate regions contains contributions from background and cytoplasmic TIRF signal, which would result in a biased intensity fraction. The intensity emitted from the nonpunctate HTLV-1 Gag-mEos2 remained approximately the same over a period of 25 min following photoconversion, which indicates that nonpunctate Gag at the membrane is not rapidly recruited into puncta and exists as a stable population over the time scale of our observation period. Because of the large intensity range present in these images, the color scale of the images in A and B was computationally saturated. The same color scale was applied to all images depicted in panels A and B. Download FIG S5, TIF file, 1.1 MB. Copyright © 2018 Eichorst et al. 2018 Eichorst et al. This content is distributed under the terms of the Creative Commons Attribution 4.0 International license.

### Measurement of Gag punctum mobility.

**FIG 2:**
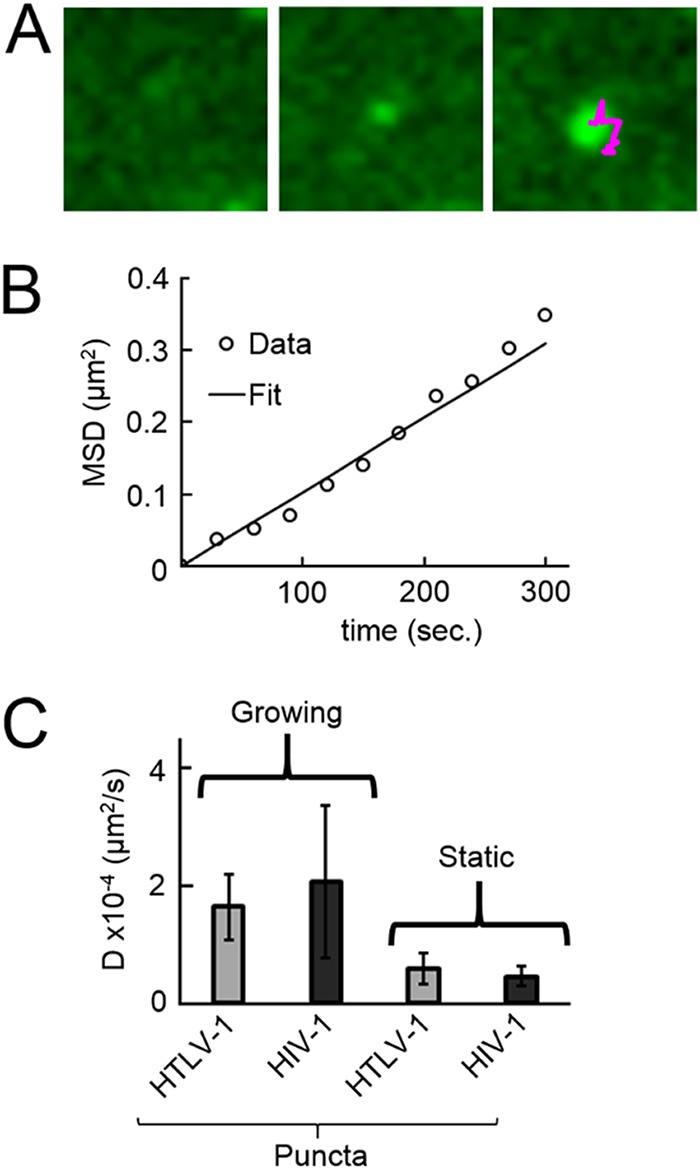
Analysis of Gag punctum biogenesis by using mean-squared displacement analysis. (A) Images of a growing punctum taken with a time interval of 2.5 min. The rightmost panel includes the punctum trajectory (magenta line) for the complete image series. (B) The mean-squared displacement (MSD) of the punctum trajectory was calculated as a function of the time difference between images. The MSD can be approximated by a linear fit, indicating that this punctum was undergoing Brownian motion while growing. The slope of the fit determines the diffusion coefficient. (C) Average diffusion coefficients (*D*) of both stationary and growing puncta produced by HTLV-1 Gag (1:3 labeled/unlabeled) and HIV-1 Gag (1:3 labeled/unlabeled).

Prior to examining the recruitment of Gag to puncta, we characterized the mobility of newly emerged puncta. Direct observation of the appearance of new puncta was achieved by imaging cells every 30 s with 488-nm and 561-nm excitation for a period of 25 min after the photoconversion step. A particle tracking algorithm identified the locations of both newly appearing and preexisting puncta from the stacks of images collected. The trajectory (magenta line in Fig. 2A) of a growing punctum found within a cell transfected with both HTLV-1 Gag-mEos2 and unlabeled HTLV-1 Gag (1:3 plasmid ratio) is shown together with a few images of the punctum at different time points. The calculated mean-squared displacement (MSD) of this trajectory is approximately linear with time (Fig. 2B), which is indicative of random (Brownian) motion. The fitted slope leads to a diffusion coefficient of 2.6 × 10^−4^ µm^2^/s. Repeated measurements (*n* = 10) of the mobility of growing HTLV-1 puncta recovered a diffusion coefficient of 1.6 × 10^−4^ µm^2^/s (Fig. 2C). Similar experiments performed on cells expressing labeled and unlabeled HIV-1 Gag (1:3 plasmid ratio) found a very similar diffusional mobility for growing HIV-1 puncta (*n* = 10) (Fig. 2C). For comparison, we determined the diffusional mobility of already existing puncta produced by HIV-1 Gag-mEos2 and HTLV-1 Gag-mEos2. Existing puncta displayed reduced mobility compared to growing puncta (Fig. 2C), which we interpret as reflecting budded particles with reduced mobility within the constricted space between the cell and the coverslip.

### Single interval imaging of Gag recruitment.

Particle tracking not only identifies the mobility of puncta, but it can also be used to study the recruitment of HIV-1 Gag to puncta (25). However, particle tracking involves repetitive imaging of the sample over extended periods of time. While observing the mobility of growing puncta, we found that the prolonged exposure to light in time-lapse imaging introduces photobleaching of the red- and green-emitting forms of mEos2, which complicates the accurate identification of the color (green intensity fraction) of the puncta.

To eliminate this problem, we reduced the number of light exposures to only two, one observation right after photoconversion to document the initial state and a second observation after a wait period of 15 min to identify newly formed puncta (referred to as single interval imaging). A newly formed punctum was defined as a punctum observed in the TIRF image of the cell collected 15 min after photoconversion which was at least 10 pixels (1.5 µm) away from any existing punctum located in the TIRF image of the same cell collected immediately after photoconversion. Because the displacement of existing puncta during the wait period is expected to be ~2.5 pixels, which is based on the measured diffusional mobility of existing puncta discussed in the previous section (Fig. 2C), the distance criterion of 10 pixels provides a safety margin to reject existing puncta. We further confirmed that the vast majority of existing puncta moved less than 10 pixels during the wait period by direct measurement (Fig. S6).

10.1128/mBio.00758-18.6 FIG S6  Displacement of Gag puncta. The displacement of existing puncta during a wait period of 15 min was determined by comparing their imaged location immediately after photoconversion and after the wait period. Only sparse regions that contained a single punctum were considered in order to clearly isolate the movement of an individual punctum. We defined a sparse area as a region with a single punctum at least 17 pixels away from its nearest neighbor. The analysis was performed on a subset of the previously imaged cells. Histograms of the measured displacements are shown for HTLV-1 Gag-mEos2 (A), HTLV-1 Gag with unlabeled HTLV-1 Gag (B), HIV-1 Gag-mEos2 (C), and HIV-1 Gag-mEos2 with unlabeled HIV-1 Gag (D). Displacements of 10 pixels or more were rare and comprised at most 11% of imaged puncta. Download FIG S6, TIF file, 0.2 MB. Copyright © 2018 Eichorst et al. 2018 Eichorst et al. This content is distributed under the terms of the Creative Commons Attribution 4.0 International license.

### Biogenesis of HIV-1 Gag puncta.

**FIG 3:**
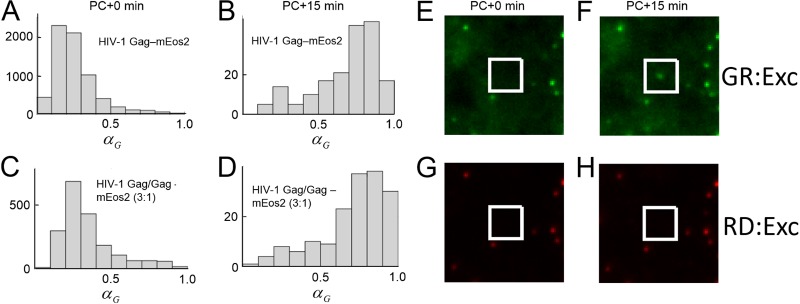
HIV-1 Gag puncta analysis following photoconversion. (A) The green color fraction of puncta from a set of HeLa cells transfected with HIV-1 Gag-mEos2 immediately after photoconversion (PC) was plotted in a histogram. (B) Histogram of the green intensity fraction for newly appearing puncta 15 min after PC. (C and D) Green color distribution of puncta from HeLa cells transfected with HIV-1 Gag-mEos2 and unlabeled HIV-1 Gag (1:3 ratio) immediately after PC (C) and of new puncta identified 15 min after PC (D). (E and G) Cropped TIRF images of a location of a future new punctum produced by HIV-1 Gag-mEos2 excited at 488 nm (E) and 561 nm (G) immediately after PC. (F and H) Cropped TIRF images of the same location taken 15 min later at 488 nm (F) and 561 nm (H). The white boxes in panels E to H indicate the locations of a newly appearing punctum recorded during the experiment, which is identified upon 488-nm excitation. GR:Exc, green excitation; RD:Exc, red excitation.

The measurement protocol described above was used to study the recruitment pathway of HIV-1 Gag by detecting the color of newly formed puncta produced by HIV-1 Gag-mEos2 in live HeLa cells. Immediately after photoconversion, the value of *α*_*G*_ corresponding to puncta located in images of cells transfected with HIV-1 Gag-mEos2 was determined (Fig. 3A). These initial values of *α*_*G*_ were centered at approximately 0.25, indicating that the HIV-1 Gag-mEos2 proteins in the puncta had been maximally converted by the 405-nm light. Newly formed puncta were identified from TIRF images taken after the wait period of 15 min. An example of a newly formed punctum is shown (Fig. 3E to H). These cropped images were taken from full frames shown in Fig. S7. The white-bordered box (20 × 20 pixel region) was initially void of puncta, but it contained a green-emitting punctum in the images taken after the wait period. The histogram of α_*G*_ for the newly appearing puncta showed a peak at ~0.8, suggesting that the new puncta contained primarily the green-emitting form of HIV-1 Gag-mEos2 (Fig. 3B). This result indicates that HIV-1 Gag-mEos2 is being recruited into puncta from the cytoplasm and is consistent with a previous report (25). However, the tail of the histogram extends to α_*G*_ values of less than 0.45, which implies that a small subset of puncta also recruit HIV-1 Gag from the plasma membrane. This observation is contrary to studies which state that HIV-1 Gag is recruited into puncta exclusively from the cytoplasm (25).

10.1128/mBio.00758-18.7 FIG S7  TIRF images of puncta containing HIV-1 Gag-mEos2 before and after PC. (A to D) Images of a HeLa cell expressing HIV-1 Gag-mEos2 collected before and after PC are scaled linearly according to the absolute maximum and minimum present in the images for each channel (Gr. Exc. and Red Exc.). (E to H) Images of the same cell in panels A to D are presented with a computationally saturated color scale in order to make features in the images more apparent. The white boxes show the locations of the new punctum depicted in Fig. 3. Download FIG S7, TIF file, 2.2 MB. Copyright © 2018 Eichorst et al. 2018 Eichorst et al. This content is distributed under the terms of the Creative Commons Attribution 4.0 International license.

In order to rule out any unwanted effects that the fluorescent tag may have on this experiment, the same experiment was repeated using a mixture of HIV-1 Gag-mEos2 and unlabeled HIV-1 Gag transfected into cells at a ratio of 1:3 (labeled plasmid/unlabeled plasmid). As shown previously in the literature, transfecting cells with this ratio of labeled and unlabeled HIV-1 Gag will result in the production of virus-like particles that contain lattice structures similar to those produced by purely unlabeled HIV-1 Gag (34). With the addition of unlabeled HIV-1 Gag in the experiment, the histograms of α_*G*_ that describe the color of photoconverted and newly formed puncta are essentially identical to the results obtained for the fully labeled Gag (Fig. 3C and D).

It has been previously reported that mEos2 can oligomerize at high concentrations (35). To address the potential impact of mEos2 association in our studies, we conducted experiments with mEos3.2 (a monomeric variant of mEos2). Using HIV-1 Gag-mEos3.2, we detected the color of newly formed puncta in live HeLa cells. As shown in Fig. S8A and B, HIV-1 Gag-mEos3.2 was primarily recruited to puncta from the cytoplasm, as evidenced by the peak in the histogram that occurred for α_*G*_ of >0.65. Furthermore, there was a minority population of new Gag puncta that were red in color (*α*_*G*_ < 0.45). Therefore, the observations using mEos3.2 are comparable to those observed with mEos2 (Fig. 3) and provide support for the conclusion that the ability of mEos2 to self-associate had no significant impact on our observations.

10.1128/mBio.00758-18.8 FIG S8  Recruitment of HIV-1 Gag-mEos3.2 and HTLV-1 Gag-mEos3.2 into puncta. (A) The green intensity fraction of puncta produced by HIV-1 Gag-mEos3.2 and present immediately after photoconversion are plotted in this histogram. (B) After a period of 15 min, the distribution of the green color fraction of new puncta containing HIV-1 Gag-mEos3.2 is similar to the corresponding histogram found for HIV-1 Gag-mEos2. (C) The histogram depicts the color of puncta present in cells transfected with HTLV-1 Gag-mEos3.2 immediately after photoconversion. (D) Following a period of 15 min, the distribution of the green color fraction of new puncta containing HTLV-1 Gag-mEos3.2 is similar to the corresponding histogram found for HTLV-1 Gag-mEos2. Download FIG S8, TIF file, 0.2 MB. Copyright © 2018 Eichorst et al. 2018 Eichorst et al. This content is distributed under the terms of the Creative Commons Attribution 4.0 International license.

### Biogenesis of HTLV-1 Gag puncta.

**FIG 4:**
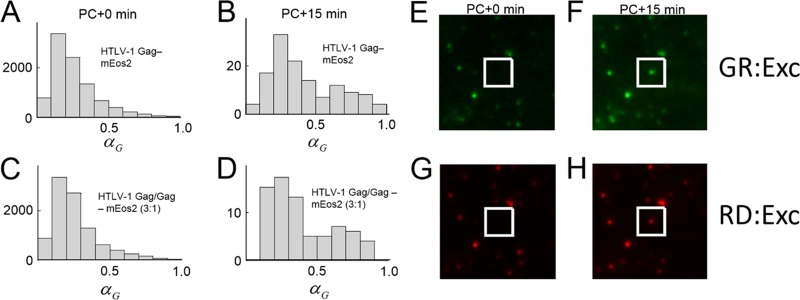
HTLV-1 Gag puncta analysis following photoconversion. (A) The green color fraction of puncta from a set of HeLa cells transfected with HTLV-1 Gag-mEos2 immediately after photoconversion (PC) was plotted in a histogram. (B) Histogram of the green intensity fraction for newly appearing puncta 15 min after photoconversion (PC). (C and D) Green color distribution of puncta from HeLa cells transfected with HTLV-1 Gag-mEos2 and unlabeled HTLV-1 Gag (1:3 ratio) immediately after PC (C) and of new puncta identified 15 min after PC (D). (E and G) Cropped TIRF images of a location of a future punctum newly produced by HTLV-1 Gag-mEos2 excited at 488 nm (E) and 561 nm (G) immediately after PC. (F and H) Cropped TIRF images of the same location taken 15 min later at 488 nm (F) and 561 nm (H). The white boxes indicate the locations of a newly appearing punctum recorded during the experiment, which fluoresces both at 488-nm (F) and 561-nm (H) excitation.

Parallel experiments examining the color of new puncta were also performed in live HeLa cells expressing HTLV-1 Gag-mEos2. In addition, cells cotransfected with a 1:3 ratio of HTLV-1 Gag-mEos2 and unlabeled HTLV-1 Gag were also measured to identify potential issues introduced by the label. The green intensity fraction *α*_*G*_ of puncta immediately after photoconversion was distributed in the red with a peak at ~0.2 (Fig. 4A and C), indicating that maximal conversion of mEos2 had been achieved. After a period of 15 min, the color fraction *α*_*G*_ of newly appearing puncta was identified and their distribution was examined (Fig. 4B and D). The majority of the newly appearing puncta were red in color (*α*_*G*_ < 0.5) for both data sets. An example of a newly appearing red punctum detected during these experiments is shown in Fig. 4E to H. The image reveals the appearance of a diffraction-limited punctum emitting more red fluorescence than green fluorescence. These cropped images were taken from full frames shown in Fig. S9. These observations suggest that the majority of new puncta are formed by preferentially recruiting from a pool of existing nonpunctate, HTLV-1 Gag-mEos2 present at the plasma membrane. The histograms of α_*G*_ also display a small shoulder near a value of 0.7 (Fig. 4B and D). Thus, it appears that there is a subpopulation of new puncta that are formed by preferential recruitment of HTLV-1 Gag from the cytoplasm.

10.1128/mBio.00758-18.9 FIG S9  TIRF images of puncta containing HTLV-1 Gag-mEos2 before and after PC. (A to D) Images of a HeLa cell expressing HTLV-1 Gag-mEos2 collected before and after PC are scaled according to the absolute maximum and minimum present in the images for each channel (Gr. Exc. and Red Exc.). (E to H) Images of the same cell in panels A to D are presented with a computationally saturated color scale in order to make features in the images more apparent. The white boxes correspond to the locations of the new punctum formed depicted in Fig. 4. Download FIG S9, TIF file, 2.6 MB. Copyright © 2018 Eichorst et al. 2018 Eichorst et al. This content is distributed under the terms of the Creative Commons Attribution 4.0 International license.

To confirm that the ability of mEos2 to oligomerize was not contributing to our observations, we monitored Gag punctum biogenesis in live HeLa cells by using HTLV-1 Gag-mEos3.2 for 15 min following photoconversion (Fig. S8C and D). New HTLV-1 Gag puncta produced were primarily red (α_*G*_ < 0.45) with a minority population of puncta that were green in color (α_*G*_ > 0.65). The observed histograms using mEos3.2 are qualitatively similar to those observed with mEos2 (Fig. 4), which indicates that the choice of label has no significant effect on the recruitment pathway.

### Analysis of Gag punctum biogenesis by time-lapse imaging.

The previously described experiments that monitored the color of newly appearing puncta from HIV-1 Gag-mEos2 and HTLV-1 Gag-mEos2 did not record the punctum over time. Thus, the possibility exists that a portion of the new puncta that appeared at the 15-min mark may not represent Gag puncta that will produce virus particles. For example, previous reports examining HIV-1 Gag labeled with the yellow fluorescent protein have shown that Gag puncta which rapidly move into and out of the excitation field in less than 1 min colocalized with proteins such as clathrin and CD63, indicating that these puncta are undergoing an endocytic process (26).

Thus, time-lapse imaging was performed on cells expressing Gag-mEos2 to provide direct confirmation of authentic punctum assembly. It is expected that a punctum proceeding through biogenesis should increase in intensity progressively over a period of several minutes. In addition, the prevalence of rapidly appearing and disappearing puncta and their influence on the previously described results can be judged by time-lapse imaging. We acquired images immediately after photoconversion for a period of 25.0 min. Images were taken every 30.0 s with an excitation power lowered by a factor of 2 to reduce the effect of photobleaching.

To examine the presence of rapidly appearing and disappearing puncta, we identified new puncta in the image taken 5 min after photoconversion using the same procedure described earlier. The length of time a new particle was present (dwell time) was identified by comparison with earlier and later images. Puncta from a total of 10 cells were analyzed to examine the dwell times of HIV-1 Gag-mEos2’s puncta (5 cells transfected with HIV-1 Gag-mEos2 and 5 cells transfected with HIV-1 Gag-mEos2 with unlabeled HIV-1 Gag at 1:3). In parallel, puncta from eight cells were analyzed to determine the dwell times of puncta produced by HTLV-1 Gag-mEos2 (four cells transfected with HTLV-1 Gag-mEos2 and four cells transfected with HTLV-1 Gag-mEos2 with unlabeled HTLV-1 Gag at 1:3).

Any new Gag puncta that were identified and tracked in these experiments were labeled short-lived if they disappeared within a minute. Analysis of tracking data revealed that only a small minority of new puncta were short-lived (Fig. S10A and B), indicating that rapidly appearing and disappearing puncta introduced no significant bias in the previously described results that examined the recruitment pathways of Gag.

10.1128/mBio.00758-18.10 FIG S10  Additional analysis of Gag puncta using particle tracking. (A and B) Particle tracking identified the dwell time of new Gag puncta by measuring the time period between appearance and disappearance of puncta from the image series. Puncta were classified as short-lived if their dwell time was less than 1 min. (A) Number of observed short-lived and total HIV-1 Gag-mEos2 puncta. (B) Number of observed short-lived and total HTLV-1 Gag-mEos2 puncta. (C and F) Recruitment of HIV-1 Gag-mEos2. Images of a cell transfected with labeled and unlabeled HIV-1 Gag (1:3 ratio) and collected with 488-nm excitation were cropped to display a growing punctum located within the white box as a function of imaging time for 488-nm excitation (C) and 561-nm excitation (D). (E) The integrated intensity of the growing puncta is shown for 488-nm excitation (green symbols) and 561-nm excitation (red symbols) as a function of time. The solid lines describe exponential charging curves with a time constant of ~6 min. (F and G) Recruitment of HTLV-1 Gag-mEos2. Images of a cell transfected with labeled and unlabeled HTLV-1 Gag (1:3 ratio) and collected with 488-nm excitation were cropped to display a growing punctum located within the white box as a function of imaging time for 488-nm excitation (F) and 561-nm excitation (G). (H) The integrated intensity of the growing puncta is shown for 488-nm excitation (green symbols) and 561-nm excitation (red symbols) as a function of time. The solid lines describe exponential charging curves with a time constant of ~5 min. Download FIG S10, TIF file, 0.3 MB. Copyright © 2018 Eichorst et al. 2018 Eichorst et al. This content is distributed under the terms of the Creative Commons Attribution 4.0 International license.

Next, a particle tracking algorithm extracted from the image stacks the locations and intensities of puncta that progressively increased in intensity. In other words, the puncta must increase in its brightness over a period of minutes to be considered a new punctum. Likewise, any punctum described as new must be well isolated from neighboring puncta during its growth so that accurate fitting and calculations of intensity can be carried out. In addition, the frames preceding the initial detection of the punctum were examined to ensure the absence of any previously existing puncta in the vicinity of the detection area. This filtering of data removed spurious and marginal events, but it also lowers the detection efficiency of new puncta. Similarly, while the excitation power was reduced to decrease photobleaching, it limited our ability to detect dimmer puncta. Thus, particle tracking experiments identified only a fraction of the newly forming puncta.

**FIG 5:**
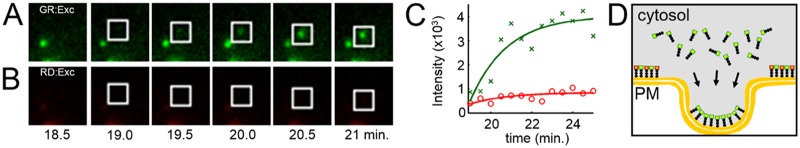
HIV-1 Gag puncta recruit Gag from the cytoplasm. (A and B) Images of a cell transfected with labeled and unlabeled HIV-1 Gag (1:3 ratio) and collected with 488-nm excitation were cropped to display a growing punctum located within the white box as a function of imaging time for 488-nm excitation (A) and 561-nm excitation (B). (C) The integrated intensity of the growing punctum is shown for 488-nm excitation (green symbols) and 561-nm excitation (red symbols) as a function of time. The solid lines describe exponential charging curves with a time constant of ~2 min. (D) Cartoon depicting the recruitment of cytoplasmic HIV-1 Gag-mEos2 into a budding particle.

An example of a growing punctum found within a cell transfected with both HIV-1 Gag-mEos2 and unlabeled HIV-1 Gag (1:3 plasmid ratio) is highlighted in the images shown in Fig. 5. The punctum appeared ~19 min after the photoconversion step and collected cytosolic (green-emitting) HIV-1 Gag-mEos2 for several minutes up to the end of the observation period (Fig. 5A). No red fluorescence was detected in this location while exciting the sample with 561-nm light (Fig. 5B). When plotted, the intensity of the punctum fluorescence emission excited with 488-nm light increased over time, while the fluorescence excited with 561-nm light remained close to the background level (Fig. 5C). This behavior is consistent with cytoplasmic Gag recruitment into the growing punctum (Fig. 5D). This is corroborated by the green intensity fraction *α*_*G*_ calculated from Fig. 5C (*α*_*G*_ = 0.8).

While photobleaching will bias the value of the green intensity fraction α_*G*_, particle tracking nevertheless provides a reasonable measure to identify the pathway of Gag recruitment. We classify growing puncta with α_*G*_ of ≥0.65 as predominantly recruiting Gag from the cytoplasm, while α_*G*_ of ≤0.45 represents predominate Gag recruitment from the plasma membrane. Any growing puncta with α_*G*_ between 0.45 and 0.65 are considered intermediate, and no preferred recruitment pathway is assigned to these puncta.

Analysis of approximately 60 HeLa cells transfected with HIV-1 Gag-mEos2 and HIV-1 Gag-mEos2 with unlabeled HIV-1 Gag identified 24 intensity traces of puncta that increased in intensity over a period of several minutes. Twelve of the traces describe puncta produced by cells transfected with HIV-1 Gag-mEos2, and the other 12 traces describe puncta produced by cells transfected with a mixture of HIV-1 Gag-mEos2 and unlabeled HIV-1 Gag.

Fourteen of the intensity traces (representing individual puncta) increased only in the channel excited by 488-nm light, similar to that seen in Fig. 5. The green color fraction of these growing puncta exceeded 0.65, indicating that Gag was recruited from the cytoplasm. In contrast, four of the intensity traces grew when excited by both 488-nm and 561-nm light, which resulted in α_*G*_ of ≤0.45, indicating a significant recruitment of membrane-bound Gag. An example with *α*_*G*_ = 0.2 is shown in Fig. S10C to E. The remaining growing puncta located in this analysis (*n* = 6) were classified as intermediate, and no recruitment pathway was assigned to them.

The rates of increase in the punctum fluorescence intensity were estimated by comparing the intensity values to exponential charging functions (e.g., Fig. 5C). With this calculation, the puncta produced in HeLa cells transfected with HIV-1 Gag-mEos2 required on the order of 5 min to reach their maximum intensity. Similarly, the puncta analyzed here that were collected from HeLa cells transfected with HIV-1 Gag-mEos2 and unlabeled HIV-1 Gag increased in intensity over a period of approximately 5 min. The estimated time for punctum growth confirmed that these puncta were not the previously reported endocytic particles that remained in the TIRF excitation field for less than 1 min, but likely puncta undergoing biogenesis.

**FIG 6:**
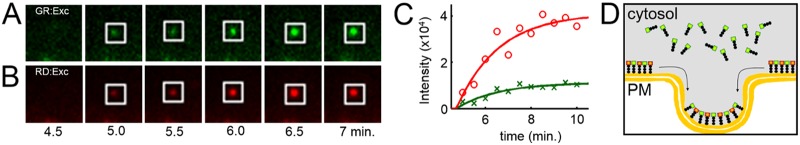
HTLV-1 Gag puncta recruit Gag from the plasma membrane. (A and B) Images of a cell transfected with labeled and unlabeled HTLV-1 Gag (1:3 ratio) and collected with 488-nm excitation were cropped to display a growing punctum located within the white box as a function of imaging time for 488-nm excitation (A) and 561-nm excitation (B). (C) The integrated intensity of the growing puncta is shown for 488-nm excitation (green symbols) and 561-nm excitation (red symbols) as a function of time. The solid lines describe exponential charging curves with a time constant of ~5 min. (D) Cartoon depicting the recruitment of nonpunctate HTLV-1 Gag-mEos2 from the plasma membrane into a budding viral particle.

The same experiments were also conducted in HeLa cells transfected with HTLV-1 Gag-mEos2 as well as HeLa cells transfected with HTLV-1 Gag-mEos2 and unlabeled HTLV-1 Gag. The particle tracking algorithm identified 23 intensity traces describing puncta that increased in intensity over time from a set of ~110 HeLa cells transfected with HTLV-1 Gag-mEos2 (11 traces) or HTLV-1 Gag-mEos2 with unlabeled HTLV-1 Gag (12 traces). Ten of the traces exhibited a time-dependent intensity increase over a few minutes with 488-nm and 561-nm excitation with green color fractions less than or equal to 0.45. An example of such a punctum with *α*_*G*_ = 0.2 is shown in Fig. 6. The low value of the green intensity fraction indicates that growing HTLV-1 puncta incorporate predominantly photoconverted HTLV-1 Gag-mEos2, which implies recruitment of nonpunctate HTLV-1 Gag bound to the plasma membrane (Fig. 6D).

Five of the collected traces resulted in α_*G*_ ≥ 0.65, which indicates that HTLV-1 Gag is likely being recruited into these puncta from the cytosol (Fig. S10F to H). The remaining detected puncta (*n* = 8) had values of α_*G*_ that were classified as intermediate, and no recruitment pathway was assigned.

The majority of traces collected for HIV-1 and HTLV-1 Gag indicate preferred recruitment from the cytoplasm and plasma membrane, respectively. This observation is consistent with the results obtained from single interval imaging (Fig. 3 and 4). We further compared the α_*G*_ values obtained from the two sets of traces (23 for HTLV-1 and 24 for HIV-1) by performing a two-tailed Mann-Whitney U test (36) with a *P* value of 0.014. Thus, the particle tracking traces provide independent evidence of statistically significant differences between HIV-1 and HTLV-1 Gag recruitment to growing puncta.

## DISCUSSION

A long-standing question regarding the assembly of enveloped viruses is how virus particle budding sites are created and how do the viral structural proteins and nucleic acid translocate to these locations on the inner leaflet of the plasma membrane. For retroviruses, the most extensive investigations have been with HIV-1 (1). Recent studies with influenza viruses have described the “budozone” as the region where viral protein self-association and subsequent virus particle budding occurs (37, 38).

Retrovirus assembly requires the orchestrated interactions of Gag-genomic RNA, Gag-Gag, and Gag-membrane in order for virus particle biogenesis to occur, leading to released particles (29). For example, Gag forms higher-order oligomers by oligomerization via interactions primarily involving the CA domain and to some degree the NC domain (21, 39–43). Once at the plasma membrane (PM), virus particle budding sites are identified and are characterized by the interaction of HIV-1 matrix (MA) domain with cholesterol-rich (44) assembly sites known as lipid rafts (45–47). Membrane binding of HIV-1 Gag is dependent upon interaction of the MA domain with phosphatidylinositol-(4,5)-bisphosphate [PI(4,5)P_2_] (48). HTLV-1 Gag has been shown to not have a preference for binding to PI(4,5)P_2_, which has implications for how HTLV-1 Gag targets the PM and identifies virus budding sites (48). Cellular factors are also recruited to the virus budding sites, resulting in budding and subsequent release of immature virus particles (21, 49, 50). The viral protease cleaves the Gag and Pol polyproteins during and shortly after the release of immature virus particles (51). The MA domain remains closely associated with the PM; the capsid (CA) domain forms a capsid shell that contains reverse transcriptase, integrase, and the nucleocapsid (NC)-coated genomic RNA (gRNA). The mature virus particle, if infectious, is capable of infecting permissive target cells (52).

In this study, we sought to conduct fundamental studies that were directed at understanding the nature of Gag punctum biogenesis—i.e., the nature of how Gag-Gag interactions result in high-ordered oligomerization and formation of Gag puncta that are capable of driving virus particle formation. In particular, we sought to compare two closely related human retroviruses that both infect CD4^+^ T cells, i.e., HIV-1 and HTLV-1. To conduct our comparative analyses, we exploited the use of a tractable model system in which the mEos2 photoconvertible fluorescence protein was fused to the Gag structural protein, and the oligomerization and accumulation of this protein over time at the membrane were quantitatively measured using TIRF. A key observation that clearly distinguishes Gag punctum biogenesis was the primary source of Gag which drives the process of particle biogenesis. In particular, we observed that HTLV-1 Gag was recruited to Gag puncta primarily from the plasma membrane, while HIV-1 Gag was recruited from the cytoplasm. These observations imply fundamental differences among retroviruses regarding the orchestration of Gag punctum biogenesis, which has important general implications for enveloped virus particle assembly.

Punctum biogenesis requires an initial seeding or nucleation of a Gag punctum, followed by growth through recruitment of additional Gag molecules. Since the detection sensitivity of our experiments is not sufficient for observing the nucleation step of biogenesis, the results shown in this paper describe the growth of the punctum after nucleation. The Gag recruitment pathway of the initial seeding of a punctum remains an open question that needs to be addressed in future experiments.

We found that accurate determination of Gag recruitment pathway(s) being utilized by a Gag labeled with the photoconvertible fluorescent protein mEos2 is technically challenging. The primary problem for live-cell imaging studies is that the green-emitting form of mEos2 loses its ability to emit fluorescence (photobleach) 5 to 10 times faster than the more commonly used green fluorescent protein (GFP) derivatives (53). In contrast, the photostability of the red-emitting form is comparable to other commonly used GFP derivatives (53). Therefore, methods such as particle tracking which repetitively image cells over long periods of time will automatically induce the loss of green fluorescence from mEos2 due to its poor photostability. Moreover, the difference in photostability between both forms of mEos2 implies that the rate of loss of green and red fluorescence will in general not be equal and complicates any subsequent quantitative analysis. For example, the fraction of green fluorescence emitted by a nascent viral particle containing Gag-mEos2, which is indicative of Gag’s recruitment pathway, cannot be obtained accurately if the amount of green and red fluorescence diminished continuously and at different rates during the imaging process. In addition, TIRF illumination efficiently excites membrane-bound Gag-mEos2 with minimal excitation of cytosolic Gag-mEos2. This causes selective photobleaching of the membrane-bound population, while the cytosolic population is essentially unaffected. Thus, the two Gag recruitment pathways are very differently impacted by photobleaching. Confounding this issue is the fact that cytoplasmic Gag experiences significant, cumulative photobleaching once it associates with the plasma membrane. The kinetics of this process are currently unknown.

These considerations prompted us to study recruitment based on the acquisition of only two sets of images. This method eliminates most of the artifacts introduced by photobleaching of mEos2 (53). In addition, the two sets of images were recorded at a higher signal level than feasible for images acquired during particle tracking experiments, further improving the ability to assess recruitment pathways. Particle tracking studies at reduced excitation powers served as controls to confirm the major findings of our imaging approach.

**FIG 7:**
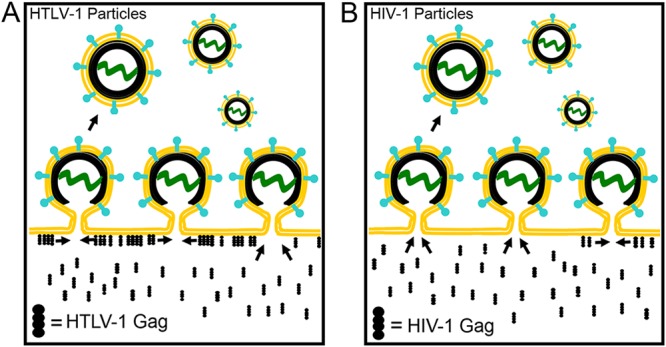
Model of HTLV-1 and HIV-1 Gag punctum biogenesis. (A) HTLV-1 Gag was found to be recruited to Gag puncta primarily from the plasma membrane (with a minority population recruited from the cytoplasm). (B) HIV-1 Gag was recruited to growing HIV-1 Gag puncta primarily from the cytoplasm.

This strategy was used to characterize HIV-1 Gag recruitment to a punctum to a degree not previously achieved (25) and contrast it to that of HTLV-1 Gag recruitment (Fig. 7). The data collected with particle tracking as well as that collected with observations at 15-min intervals allowed us to determine that a subset of HTLV-1 Gag recruitment occurred from the cytoplasm and not from the inner leaflet of the plasma membrane. In particular, histograms describing the color of new HTLV-1 Gag puncta formed after 15 min revealed a minor green population, indicating that HTLV-1 Gag could be recruited to Gag puncta from the cytoplasm. Likewise, parallel measurements carried out on cells transfected with HIV-1 Gag-mEos2 indicated that a minor population of the recorded puncta were red and therefore were likely recruiting HIV-1 Gag-mEos2 from plasma membrane.

Previously published data that examined the recruitment of HIV-1 Gag into budding sites indicate that almost all puncta produced by HIV-1 Gag-mEos2 grow by recruitment from the cytosol (Fig. 5F of reference 25). It is possible that some of the data points with low values reflect the existence of a minority population of puncta with a different recruitment pathway, similar to the observations reported here. However, the published report provided no quantitative assessment of the data. The improvements described in this study enable us to link the measured green color fraction with the probable pathway of Gag recruitment. Our data not only confirm that HIV-1 Gag is predominantly recruited into budding sites by cytosolic recruitment but also provide direct experimental evidence for a minority population with predominant recruitment of Gag from the plasma membrane.

The relative contribution of Gag recruitment from the cytosol and the plasma membrane of a punctum is related to the molar ratios of converted and unconverted mEos2 within the punctum. However, this information is currently not accessible from the green intensity ratio *α*_*G*_ without a detailed model of the photophysics of mEos2 in a punctum. For example, fluorescence resonance energy transfer (FRET) between the two states of mEos2 within a punctum reduces the fluorescence from the green state while increasing the fluorescence of the red state of mEos2, which leads to a lowering of α_*G*_ compared to α_*G*_ without FRET. Because no general modeling of the fluorescence intensities in the presence of FRET involving multiple acceptors and donors exists, no quantitative mapping of *α*_*G*_ was attempted. Instead, we used *α*_*G*_ as a convenient parameter to classify puncta. Specifically, *α*_*G*_ < 0.45 and *α*_*G*_ > 0.65 were used to mark puncta with predominant recruitment of Gag from the plasma membrane and cytosol, respectively, and were chosen based on reference measurements of mEos2 in the unconverted and fully photoconverted state (Fig. 3A and C and 4A and C; also see Fig. S2 in the supplemental material).

It is formally possible that the overall expression level of HIV-1 Gag and HTLV-1 Gag in cells may influence the recruitment pathway of Gag into puncta. The expression levels of HIV-1 and HTLV-1 Gag-mEos2 in the cells examined here are anticipated to be similar. This conclusion is based on a previous study which indicated that the range of observable cytoplasmic concentrations of the codon-optimized HTLV-1 and HIV-1 Gag constructs labeled with enhanced GFP (EGFP) were essentially identical (54). Unfortunately, quantification of the cytosolic concentration of Gag in parallel with our TIRF-based studies remains technically very challenging. It will be interesting to correlate *α*_*G*_ of newly formed puncta with the cytoplasmic Gag concentration in future experiments to directly investigate potential correlations between cytoplasmic Gag concentration and the recruitment pathway of Gag into puncta.

In these studies, we acknowledge that the use of a codon-optimized Gag as a surrogate may not fully recapitulate the expression of authentic Gag in the context of integrated proviral DNA. However, many aspects of our results obtained using codon-optimized HIV-1 Gag (e.g., primary recruitment of Gag from the cytoplasm) match well with the results of other investigators, which provides a degree of independent validation of our findings. Although a fluorescently labeled full-length expression vector of HTLV-1 Gag has not been published, several groups have examined the distribution of HTLV-1 Gag in cells using fluorescently labeled antibodies (16, 55, 56). In these reports, HTLV-1 Gag was distributed dominantly along the plasma membrane of the cell, which is consistent with our current and previous findings. Finally, our use of paired codon-optimized Gag expression constructs provides an internal control that minimizes differences in parallel experiments with HTLV-1 and HIV-1 Gag proteins. Ultimately, our studies provide a stepping stone toward ultimate validation of these findings with authentic Gag in the context of integrated proviral DNA.

Finally, it is possible that the biogenesis of HTLV-1 Gag puncta by recruitment of Gag along the plasma membrane is a more efficient mode of particle production during cell-cell contacts. Cell-free particle infectivity for HTLV-1 is low, and recent observations have indicated that only a portion of mature HTLV-1 particles have intact cores (57). Infectious HTLV-1 spread is more heavily dependent upon cell-to-cell transmission than that of HIV-1. Likewise, in studies of HTLV-1 in T cells using electron microscopy, there is an accumulation of Gag at cell-cell junctions in the form of nonpunctate and punctate Gag (20, 58, 59). On the basis of these observations, it is formally possible that the biogenesis of HTLV-1 Gag puncta by recruitment of Gag along the plasma membrane is a more efficient mode of particle production during cell-cell contacts. Further experiments are needed to test whether the recruitment of Gag into particles at the plasma membrane is sufficient or required for the biogenesis of infectious viral particles at cell-cell junctions.

These results support the conclusion that although HTLV-1 Gag recruitment is primarily from the plasma membrane (Fig. 7A) and HIV-1 Gag is recruited into puncta dominantly from the cytosol (Fig. 7B), the recruitment of Gag may, in part, depend on the local environment of the site of Gag punctum biogenesis. Cytoplasmic Gag would likely be readily accessible to all Gag puncta. However, to explain the existence of puncta that predominantly recruit plasma membrane-bound Gag, we have to postulate not only the existence of puncta surrounded by nonpunctate Gag but also that the punctum preferentially incorporates nonpunctate Gag from the membrane over cytosolic Gag. Future studies are needed to investigate the possible role that high concentrations of nonpunctate Gag at the plasma membrane may have in the formation of viral particles associated with either HIV-1 or HTLV-1.

## MATERIALS AND METHODS

### Instrumentation and imaging experimental setup.

All experiments were conducted using a Zeiss Axio Observer Z1 microscope equipped with a 100× 1.45-numerical-aperture (NA) oil immersion objective (Zeiss, Germany). Images were collected using an Andor electron-multiplying charge-coupled-device (EMCCD) model DV887 camera (Andor, Belfast, Ireland). A 405-nm laser (model 405-06-01-0100-100; Cobalt, Sweden), a 488-nm laser (Coherent), and a 561-nm laser (model 0561-04-01-0100-700; Cobalt, Sweden) were used for illumination in the total internal reflection fluorescence microscopy (TIRF) mode. The color and intensity of light incident on the sample were controlled with an acousto-optic tunable filter (AOTF) (AA Opto Electronic, France). The excitation light and emission light were separated with a multicolor dichroic cube (model TRF89901-ET; Chroma, Bellows Falls, VT). HeLa cells transfected with HIV-1 Gag-mEos2 or HTLV-1 Gag-mEos2 were imaged with each of the three lasers (405 nm, 488 nm, and 561 nm) using the multicolor dichroic cube to separate excitation from emission.

### Cells and plasmid DNAs.

HeLa cells (ATCC, Manassas, VA) were transfected with GenJet following the manufacturer’s protocol. For measurements, cells were maintained in L15 CO_2_-independent medium (Invitrogen, Waltham, MA) supplemented with 10% fetal calf serum. The temperature on the microscope stage was maintained at 37°C with a stage heater (model ASI 400; NevTek, Williamsville, VA). HeLa cells were measured 6 to 20 h after transfection. The HIV-1 Gag-EYFP plasmid (60) was modified to contain the mEos2 protein between the NotI and EcoRI restriction sites. The enhanced yellow fluorescent protein (EYFP) in expression construct pHTLV-1 Gag-EYFP described previously (27, 61) was exchanged with the mEos2 sequence by using a subcloning kit. For the cell viability assays, 50 µl of NucBlue (Fisher Scientific, Waltham, MA) was added directly to 300 µl of the L15 medium during imaging experiments.

### Calculation of Gag punctum intensity.

The initial locations of all puncta were determined from images collected by TIRF using the feature-locating function (mpretack) developed by the Kilfoil group (62). Once the location was established, the puncta and region surrounding the puncta were cropped, and segmentation with k-means clustering was performed to remove background. The edge of the cropped image frame was then masked to avoid contributions from any nearby puncta when fitting. The cropped image was fit to a two-dimensional (2D) Gaussian function to determine the integrated intensity of the punctum. In some cases, there was insufficient intensity present to accurately fit the data to a Gaussian function. This situation occurred, for example, in the absence of photoconversion, which resulted in a prominent punctum observed with 488-nm light but no feature in the corresponding cropped image collected with 561-nm excitation. Instead of fitting these areas of low intensity, the intensities in an area equal to the average size of the punctum in our image (3 × 3 pixels) were summed and used in subsequent calculations. Any shift in the image that occurred during subsequent measurements of cells transfected with HIV-1 or HTLV-1 Gag-mEos2 was corrected by an affine image transformation before any calculations were performed.

### Gag punctum tracking and analysis.

The positions of growing puncta were determined by MatLab implementation of a previously developed algorithm (62). The output of this software was then further processed with custom filters written in MatLab (MathWorks, Natick, MA) to locate only those puncta that progressively gained intensity over time. For the experiments such as the recruitment study involving long wait periods, the locations of puncta in static images were identified using the mpretrack function, which was implemented in MatLab. All supplemental functions for calculating distances to neighboring puncta were custom written in MatLab. The time-dependent intensity *I*(*t*) of growing puncta was fit to an exponential charging curve defined by *I* (*t*) = *A*[1 − exp(−*t*/τ)], where *A* is the amplitude and τ is the time constant. Because reduction of the excitation power reduced the signal-to-background ratio of particle tracking, the green intensity fraction was calculated after subtraction of the background signal determined from the image frames preceding punctum detection.
