# Supplementary figures and images for: Distinct Pathway of Human T-Cell Leukemia Virus Type 1 Gag Punctum Biogenesis Provides New Insights into Enveloped Virus Assembly
Source: mBio. 2018 Sep 4;9(5):e00758-18. doi: 10.1128/mBio.00758-18 (PMC6123448; doi:10.1128/mBio.00758-18)

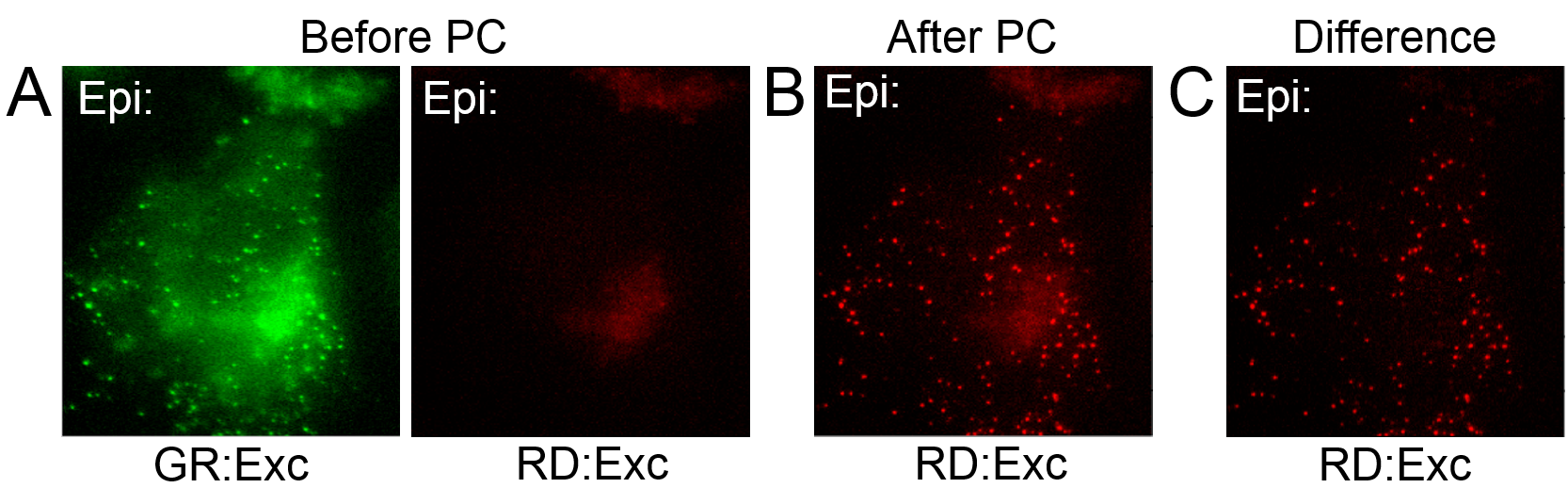

Supplement: FIG S1 [file mbo004184030sf1.tif]

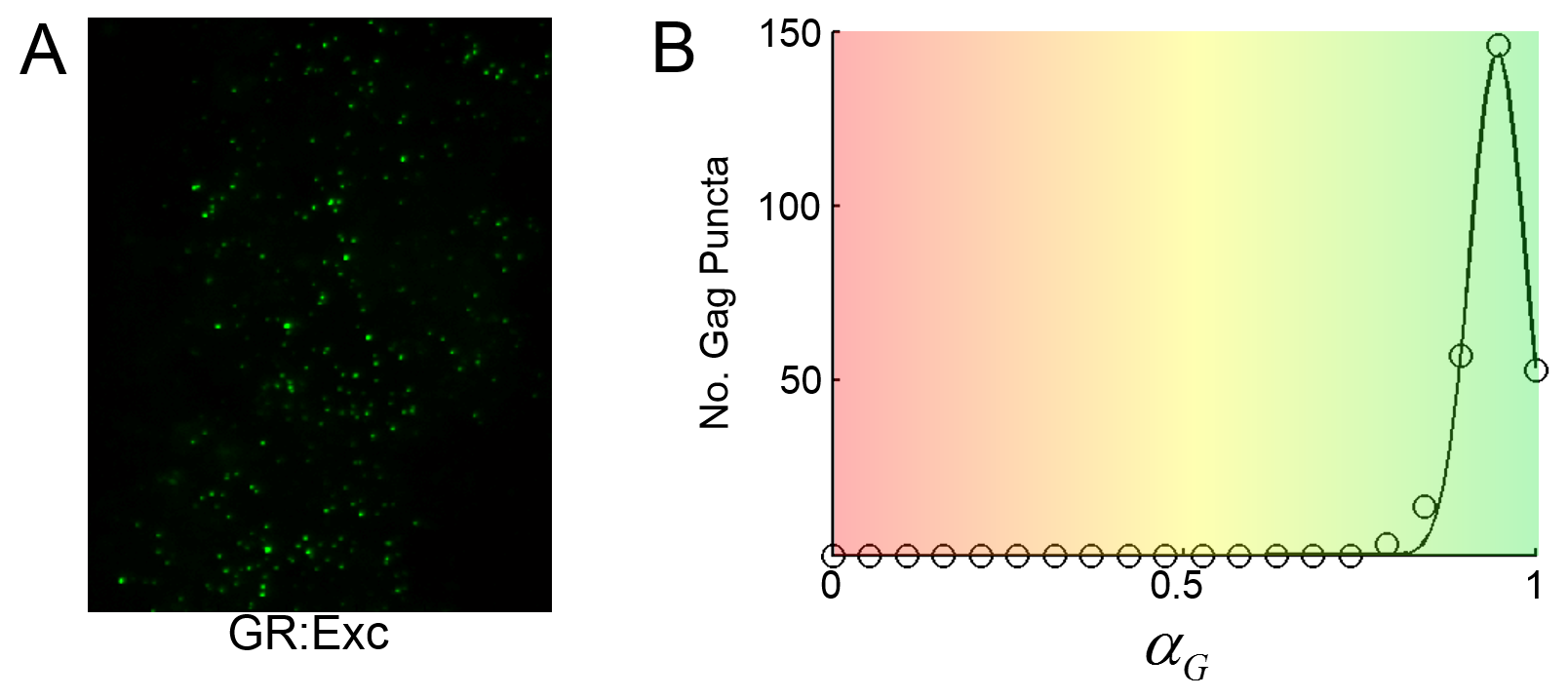

Supplement: FIG S2 [file mbo004184030sf2.tif]

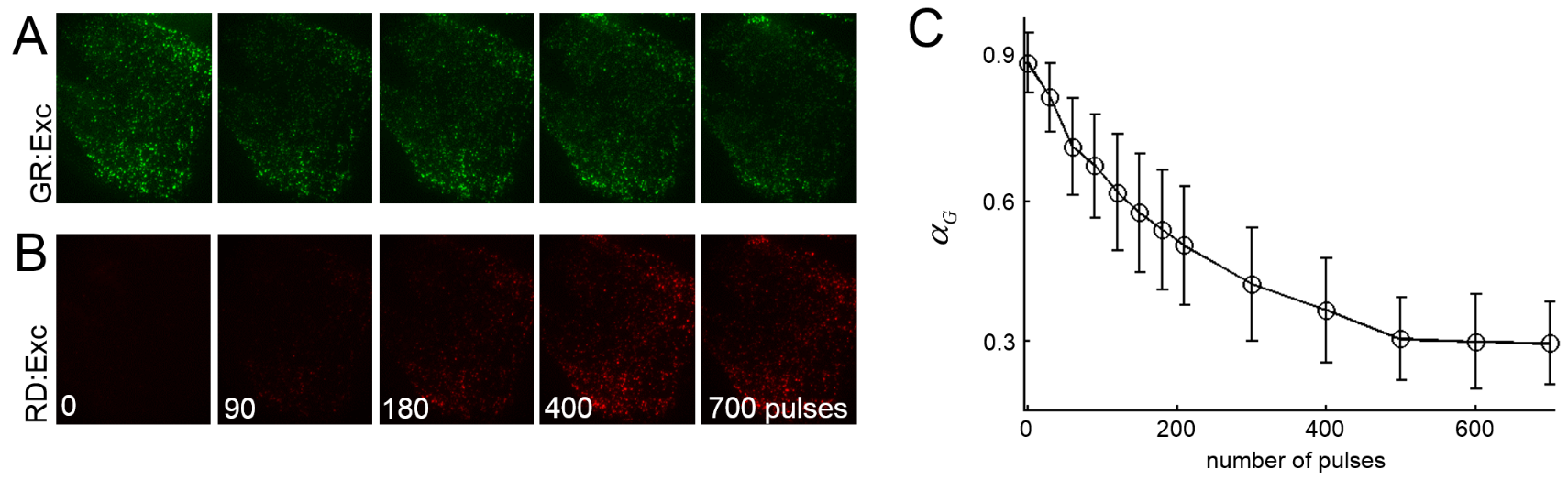

Supplement: FIG S3 [file mbo004184030sf3.tif]

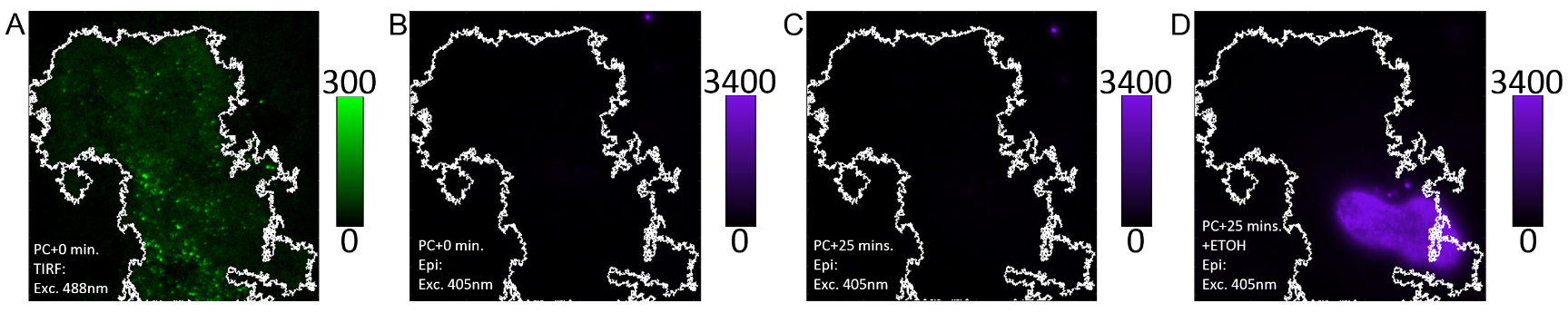

Supplement: FIG S4 [file mbo004184030sf4.tif]

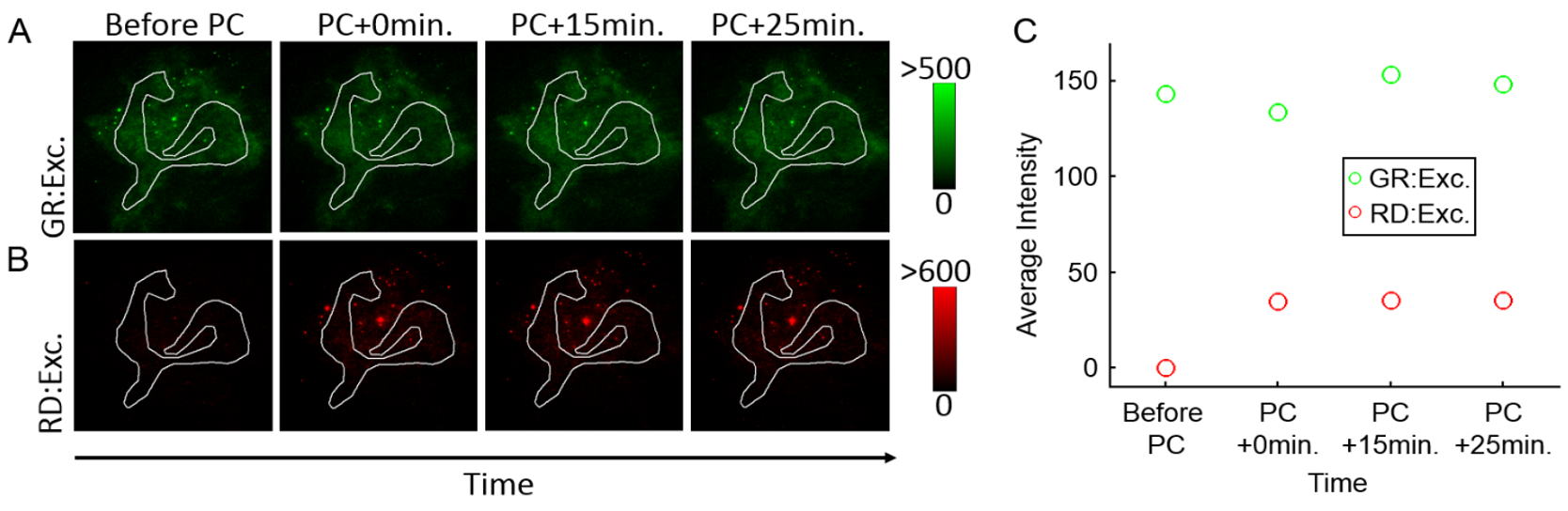

Supplement: FIG S5 [file mbo004184030sf5.tif]

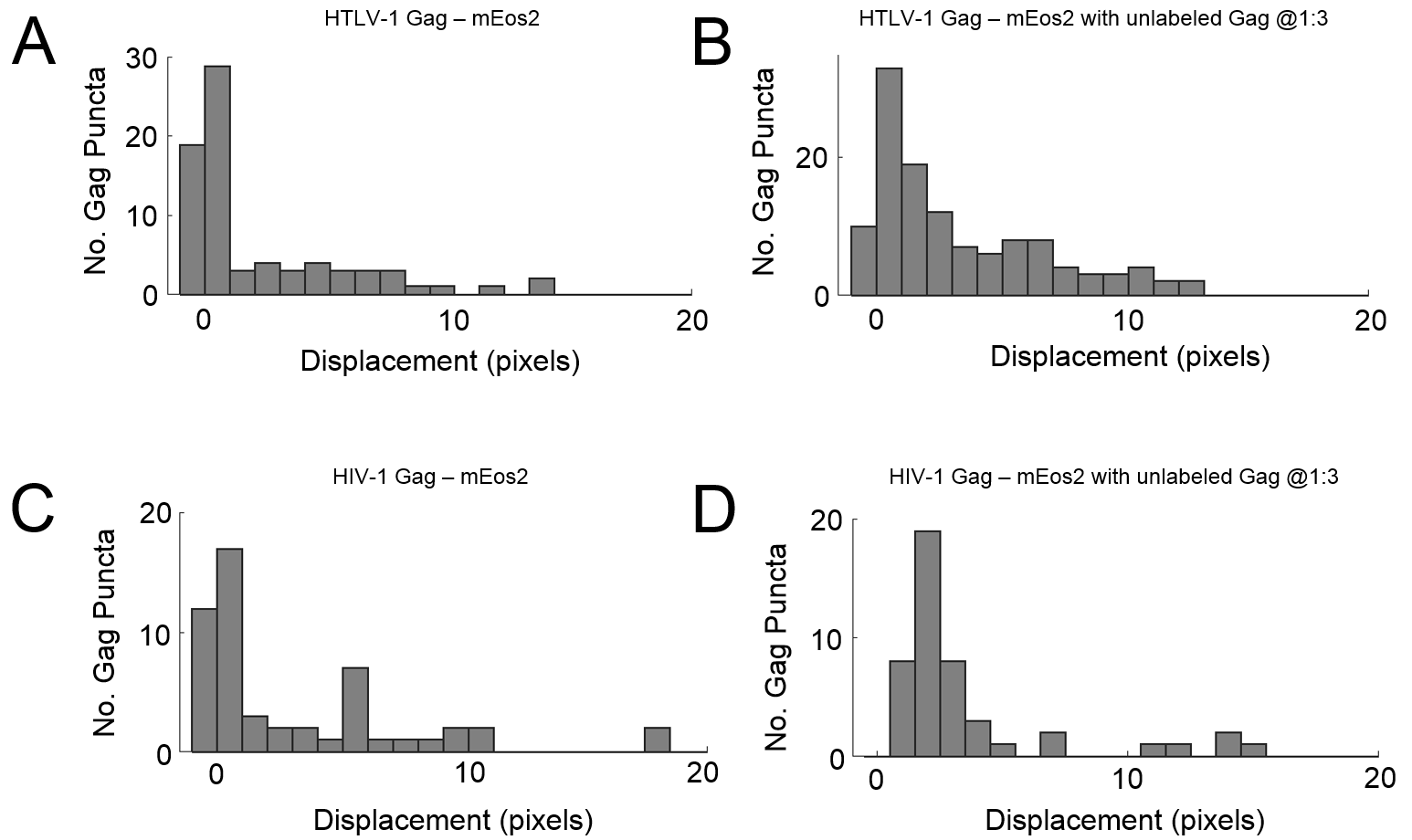

Supplement: FIG S6 [file mbo004184030sf6.tif]

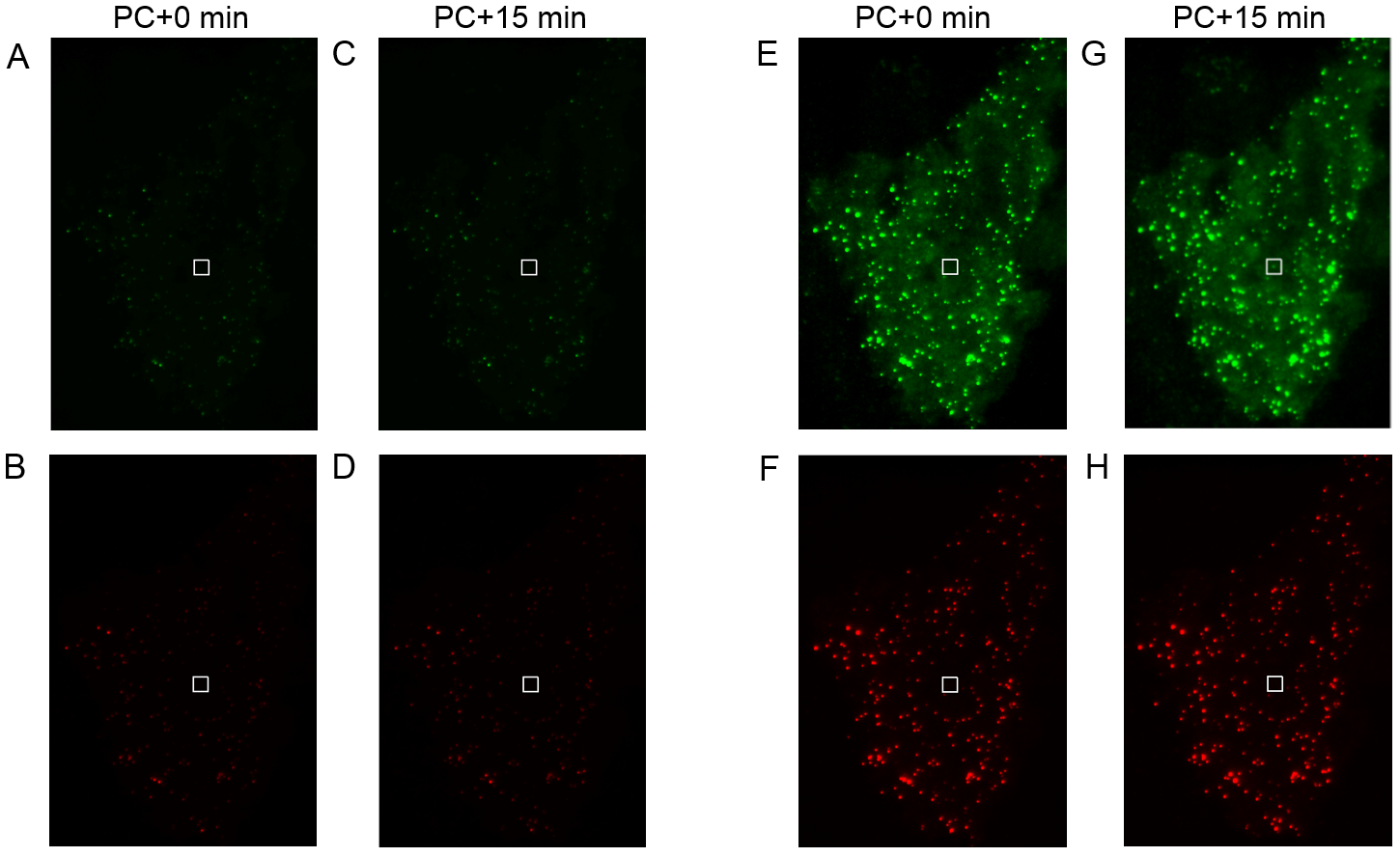

Supplement: FIG S7 [file mbo004184030sf7.tif]

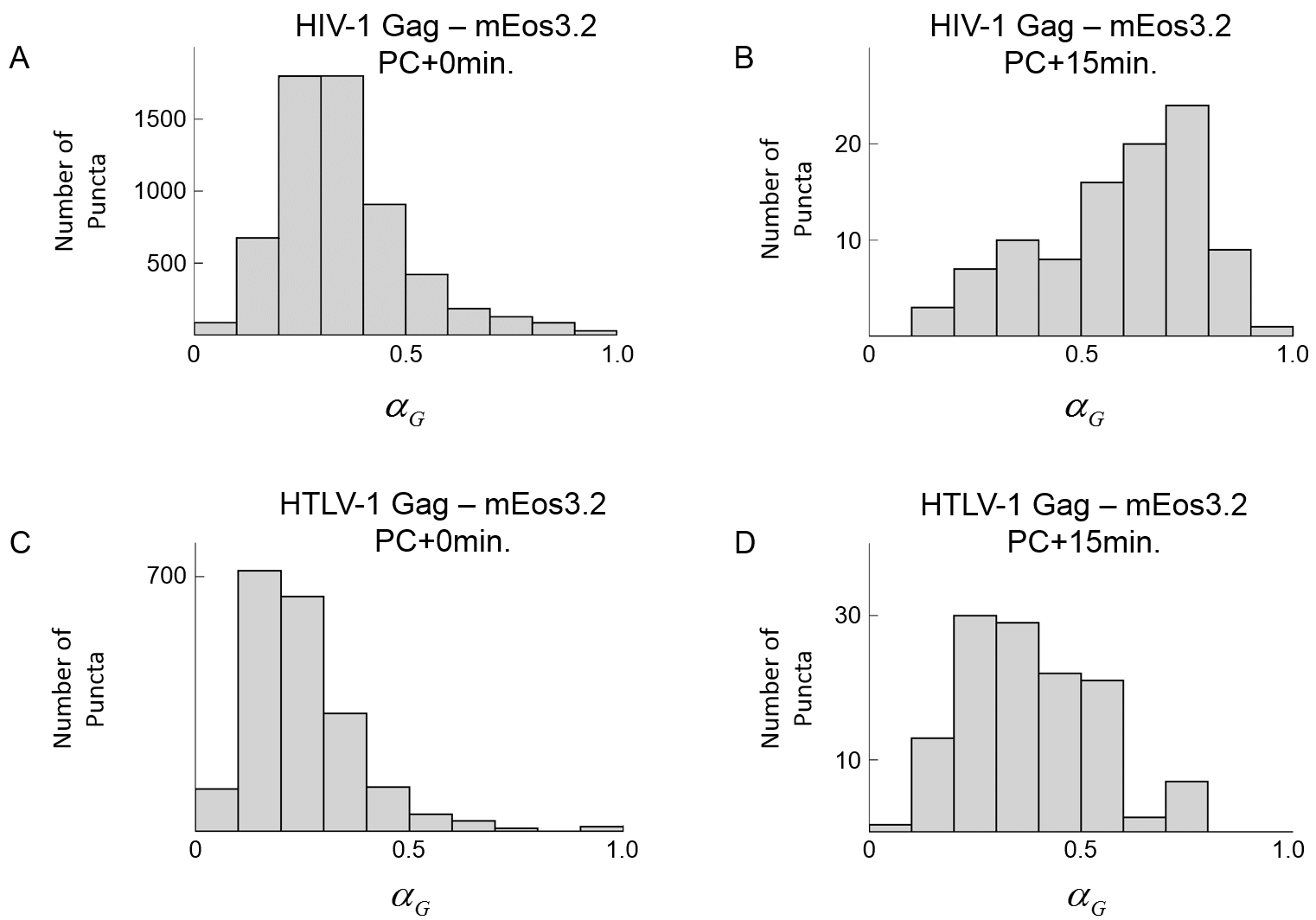

Supplement: FIG S8 [file mbo004184030sf8.tif]

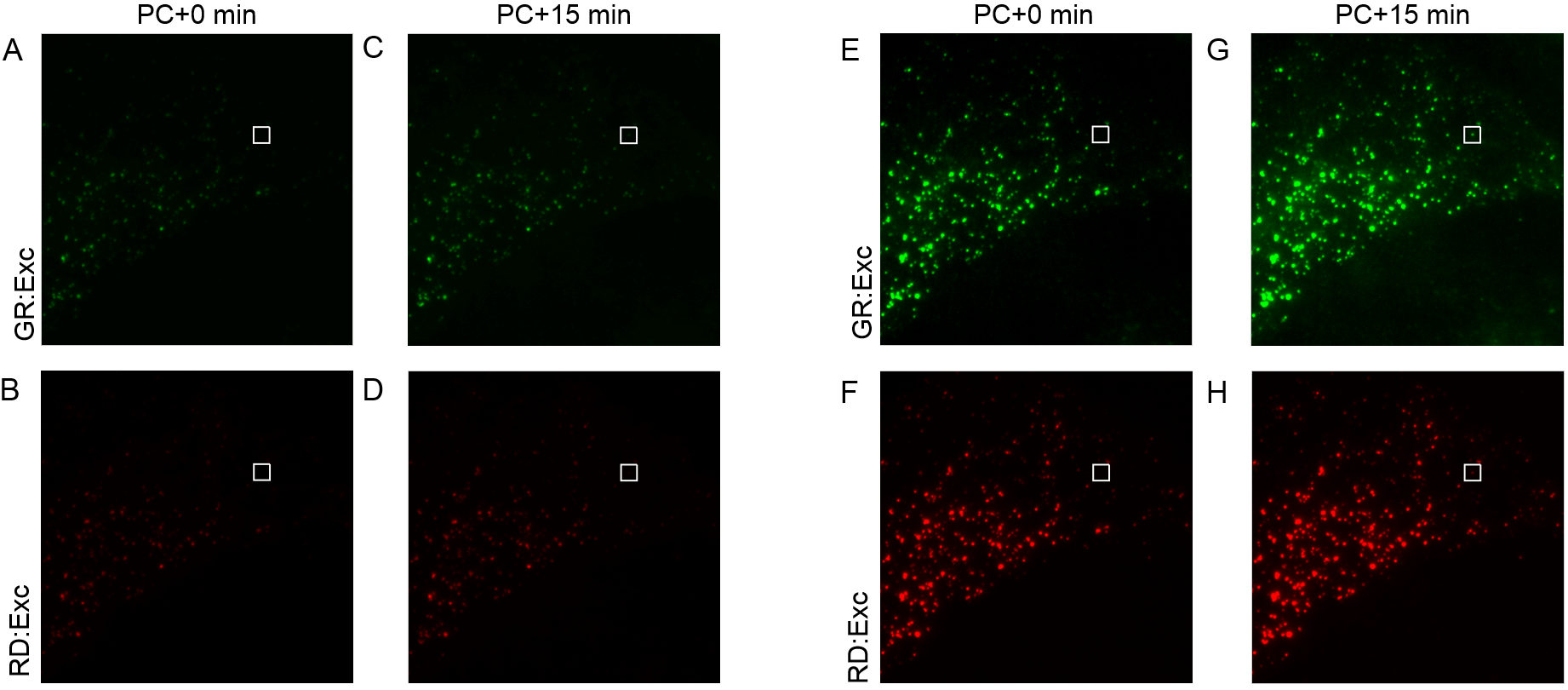

Supplement: FIG S9 [file mbo004184030sf9.tif]

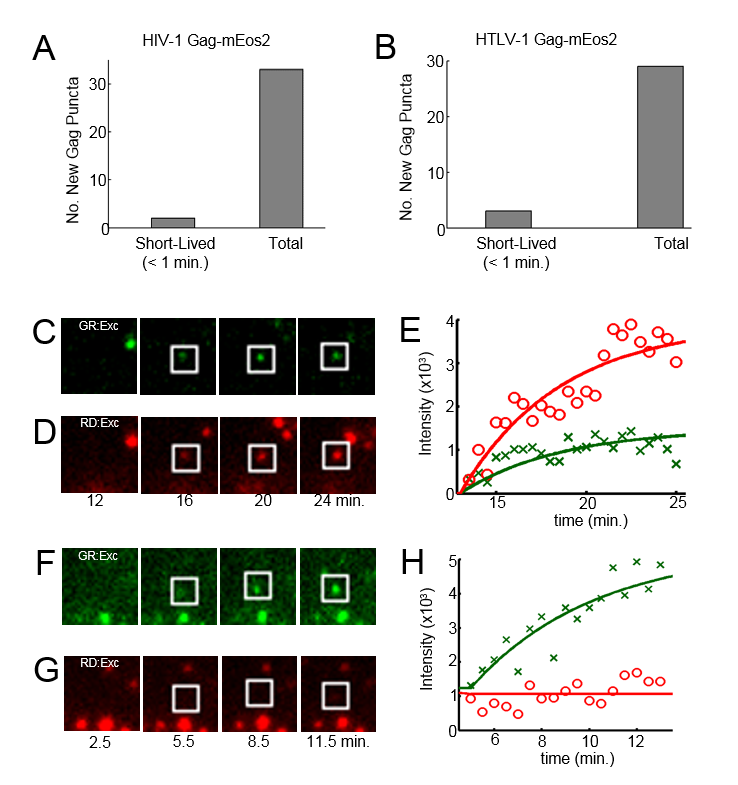

Supplement: FIG S10 [file mbo004184030sf10.tif]
